# Supplementary material for: An overview of the characteristics and quality assessment criteria in systematic review of pharmacoeconomics
Source: PLoS One. 2021 Feb 8;16(2):e0246080. doi: 10.1371/journal.pone.0246080 (PMC7870091; doi:10.1371/journal.pone.0246080)
Supplement: S1 Text — (DOCX) [file pone.0246080.s001.docx]

**S1 Text: Search strategies for English and Chinese databases.**

| **Database** | **Search strategies** |
| --- | --- |
| PubMed | #1Cost-Benefit Analysis[mesh] OR Cost Benefit Analysis[tiab] OR Cost Utility Analysis[tiab] OR Cost Effectiveness Analysis[tiab] OR Cost Analysis[tiab] OR Economic Evaluation[tiab]  #2"Review Literature as Topic"[Mesh] OR “systematic review”[tiab]  #3 #1 AND #2 |
| EMbase (Ovid) | #1 Cost-Benefit Analysis [subject headings] OR Cost Benefit Analysis[tiab] OR Cost Utility Analysis[tiab] OR Cost Effectiveness Analysis[tiab] OR Cost Analysis[tiab] OR Economic Evaluation[tiab]  #2 systematic review[subject headings] OR systematic review[tiab]  #3 #1 AND #2 |
| NHS EED (Ovid) | #1 Cost-Benefit Analysis [Map Term] OR Cost Benefit Analysis[All Fields] OR Cost Utility Analysis[All Fields] OR Cost Effectiveness Analysis[All Fields] OR Cost Analysis[All Fields]OR Economic Evaluation[All Fields]  #2 systematic review[mp.] OR systematic review [All Fields]  #3 #1 AND #2 |
| The Cochrane Library  (CENTRAL) | #1 Cost-Benefit Analysis [ti,ab,kw] OR Cost Benefit Analysis[ti,ab,kw] OR Cost Utility Analysis[ti,ab,kw] OR Cost Effectiveness Analysis[ti,ab,kw] OR Cost Analysis[ti,ab,kw]  #2 Economic Evaluation[ti,ab,kw]  #3 #1 OR #2 |
| HTA Database | #1 Cost-Benefit Analysis [Map Term] OR Cost Benefit Analysis[All Fields] OR Cost Utility Analysis[All Fields] OR Cost Effectiveness Analysis[All Fields] OR Cost Analysis[All Fields]OR Economic Evaluation[All Fields]  #2 systematic review[mp.] OR systematic review [All Fields]  #3 #1 AND #2 |
| CNKI | #1 SU= "经济学"+"成本效果"+"成本效益"+"成本效用"  #2 SU="系统评价" OR TI="系统评价" OR AB="系统评价"  #3 #1 AND #2 |
| WanFang Data | #1主题:"经济学"+"成本效果"+"成本效益"+"成本效用"  #2主题: "系统评价"  #3 #1*#2 |
| VIP | #1 题名或关键词=经济学 或 成本效果 或 成本效益 或 成本效用  #2 题名或关键词=系统评价  #3 #1 *#2 |
| CBM | #1 中文摘要：“经济学” OR “成本效果” OR “成本效益” OR “成本效用”  #2 中文标题：“系统评价”  #3 中文摘要：“系统评价”  #4 #1 AND #2 AND #3 |

HTA, Health technology assessment; CNKI, Chinese National Knowledge Infrastructure; VIP, VIP Chinese Science & Technology Periodicals; CBM, Chinese Biomedical Literature Database.
