# Supplementary material for: An overview of the characteristics and quality assessment criteria in systematic review of pharmacoeconomics
Source: PLoS One. 2021 Feb 8;16(2):e0246080. doi: 10.1371/journal.pone.0246080 (PMC7870091; doi:10.1371/journal.pone.0246080)
Supplement: S2 Text — (DOCX) [file pone.0246080.s002.docx]

**S2 Text: The references of included 165 studies.**

1. Jordan R, Gold L, Hyde C, Cummins C. Antiretroviral therapy for HIV infection in patients naive to prior treatment: a systematic review of effectiveness and cost-effectiveness. Health Technol Assess. 2000;(2):1-135.
2. Lister-Sharp D, McDonagh MS, Khan KS, Kleijnen J. A rapid and systematic review of the effectiveness and cost-effectiveness of the taxanes used in the treatment of advanced breast and ovarian cancer. Health Technol Assess. 2000;4(17):1-113. https://doi.org/ 10.3310/hta4170 PMID: 11074389.
3. Forbes C, Shirran L, Bagnall AM, Duffy S, ter Riet G. A rapid and systematic review of the clinical effectiveness and cost-effectiveness of topotecan for ovarian cancer. Health Technol Assess. 2001;5(28):1-110. https://doi.org/ 10.3310/hta5280 PMID: 11701100.
4. O'Meara S, Riemsma R, Shirran L, Mather L, ter Riet G. A rapid and systematic review of the clinical effectiveness and cost-effectiveness of orlistat in the management of obesity. Health Technol Assess. 2001;5(18):1-81. https://doi.org/ 10.3310/hta5180 PMID: 11399238.
5. Creese A, Floyd K, Alban A, Guinness L. Cost-effectiveness of HIV/AIDS interventions in Africa: a systematic review of the evidence. Lancet. 2002;359(9318):1635-1643. https://doi.org/ 10.1016/S0140-6736(02)08595-1 PMID: 12020523.
6. Forbes C, Wilby J, Richardson G, Sculpher M, Mather L, Riemsma R. A systematic review and economic evaluation of pegylated liposomal doxorubicin hydrochloride for ovarian cancer. Health Technol Assess. 2002;6(23):1-119. https://doi.org/ 10.3310/hta6230 PMID: 12433314.
7. Hancock S, Wake B, Hyde C. Fludarabine as first line therapy for chronic lymphocytic leukaemia Database of Abstracts of Reviews of Effects. 2002;(2):1-60. https://doi.org/ PMID: DARE-12004008031
8. Lewis R, Bagnall AM, King S, Woolacott N, Forbes C, Shirran L, et al. The clinical effectiveness and cost-effectiveness of vinorelbine for breast cancer: a systematic review and economic evaluation. Health Technol Assess. 2002;6(14):1-269. https://doi.org/ 10.3310/hta6140 PMID: 12583816.
9. Robinson M, Ginnelly L, Sculpher M, Jones L, Riemsma, Palmer S, et al. A systematic review update of the clinical effectiveness and cost-effectiveness of glycoprotein IIb/IIIa antagonists. Health Technol Assess. 2002;6(25):1-160. https://doi.org/ 10.1016/0735-6757(91)90003-3 PMID: 12583818.
10. Boland A, Dundar Y, Bagust A, Haycox A, Hill R, Mota RM, et al. Early thrombolysis for the treatment of acute myocardial infarction: a systematic review and economic evaluation Health Technology Assessment. 2003;(15):1-136. https://doi.org/ PMID: DARE-12003008341
11. Penaloza Hidalgo B, Knight T, Burls A. A systematic review of effectiveness and cost effectiveness of tacrolimus ointment for topical treatment of atopic dermatitis in adults and children. Health Technology Assessment Database. 2003;(2):1-96.
12. Segal JB, Bolger DT, Jenckes MW, Krishnan JA, Streiff MB, Eng J, et al. Outpatient therapy with low molecular weight heparin for the treatment of venous thromboembolism: a review of efficacy, safety, and costs American Journal of Medicine. 2003;(4):298-308. https://doi.org/ PMID: DARE-12003002024
13. Bridle C, Palmer S, Bagnall A, Darba J, Duffy S, Sculpher M, et al. A rapid and systematic review and economic evaluation of the clinical and cost-effectiveness of newer drugs for treatment of mania associated with bipolar affective disorder.2004. NIHR Health Technology Assessment programme: Executive Summaries: NIHR Journals Library.
14. Brinsmead R, Hill S, Walker D. Are economic evaluations of vaccines useful to decision-makers? Case study of Haemophilus influenzae type b vaccines. Pediatric Infectious Disease Journal. 2004;23(1): 32-37. https://doi.org/ 10.1097/01.inf.0000105104.39284.a3
15. Dixon S, McKeen E, Tabberer M, Paisley S. Economic evaluations of treatments for systemic fungal infections: a systematic review of the literature. Pharmacoeconomics. 2004;22(7):421-433. https://doi.org/ 1170-7690/04/0007-0421/$31.00/0 PMID: 15137881.
16. Jones L, Griffin S, Palmer S, Main C, Orton V, Sculpher M, et al. Clinical effectiveness and cost-effectiveness of clopidogrel and modified-release dipyridamole in the secondary prevention of occlusive vascular events: a systematic review and economic evaluation. Health Technol Assess. 2004;8(38):iii-iv, 1-196. https://doi.org/ 10.3310/hta8380 PMID: 15461876.
17. Main C, Palmer S, Griffin S, Jones L, Orton V, Sculpher M, et al. Clopidogrel used in combination with aspirin compared with aspirin alone in the treatment of non-ST-segment-elevation acute coronary syndromes: a systematic review and economic evaluation. Health Technol Assess. 2004;8(40):1-141. https://doi.org/ 10.3310/hta8400 PMID: 15461878.
18. Sun Xin YH, Wang Li, Orlewska E, Li You-ping. Cost-effectiveness of Treatment of Chronic Hepatitis in China: A Systematic Review. Chinese J Evidence-based Medicine. 2005;5(11):833-845. https://doi.org/ 10.3969/j.issn.1672-2531.2005.11.006 PMID:
19. Wilby J, Kainth A, Hawkins N, Epstein D, McIntosh H, McDaid C, et al. Clinical effectiveness, tolerability and cost-effectiveness of newer drugs for epilepsy in adults: A systematic review and economic evaluation. Health Technology Assessment. 2005;9(15):1-157. https://doi.org/ 10.3310/hta9150
20. Ying X. Health technology assessment of three kinds of drugs in the treatment of benign prostatic hyperplasia.scholarly journal. [Master Thesis]. Sichuan University.
21. Brown TJ, Hooper L, Elliott RA, Payne K, Webb R, Roberts C, et al. A comparison of the cost-effectiveness of five strategies for the prevention of non-steroidal anti-inflammatory drug-induced gastrointestinal toxicity: a systematic review with economic modelling. Health Technol Assess. 2006;10(38): 1-183. PMID: 17018227.
22. Chen YF, Jobanputra P, Barton P, Jowett S, Bryan S, Clark W, et al. A systematic review of the effectiveness of adalimumab, etanercept and infliximab for the treatment of rheumatoid arthritis in adults and an economic evaluation of their cost-effectiveness. Health Technology Assessment. 2006;10(42):1-138. https://doi.org/ 10.3310/hta10420.
23. Loveman E, Green C, Kirby J, Takeda A, Picot J, Payne E, et al. The clinical and cost-effectiveness of donepezil, rivastigmine, galantamine and memantine for Alzheimer's disease. Health Technology Assessment. 2006;10(1):1-160. https://doi.org/ 10.3310/hta10010.
24. Pandor A, Eggington S, Paisley S, Tappenden P, Sutcliffe P. The clinical and cost-effectiveness of oxaliplatin and capecitabine for the adjuvant treatment of colon cancer: systematic review and economic evaluation. Health Technol Assess. 2006;10(41):1-185. https://doi.org/ 10.3310/hta10410 PMID: 17049138.
25. Woolacott N, Hawkins N, Mason A, Kainth A, Khadjesari Z, Bravo Vergel Y, et al. Etanercept and efalizumab for the treatment of psoriasis: a systematic review Health Technology Assessment Database. 2006;10(46):1-233. https://doi.org/ 10.3310/hta10460.
26. Xin S. Effectiveness and cost-effectiveness evaluation of antiviral drugs in the treatment of HBeAg-positive chronic hepatitis B.scholarly journal. [Master Thesis]. Sichuan University.
27. Collins R, Fenwick E, Trowman R, Perard R, Norman G, Light K, et al. A systematic review and economic model of the clinical effectiveness and cost-effectiveness of docetaxel in combination with prednisone or prednisolone for the treatment of hormone-refractory metastatic prostate cancer. Health Technology Assessment. 2007;11(2):1-97. https://doi.org/ 10.3310/hta11020.
28. Connock M, Juarez-Garcia A, Jowett S, Frew E, Liu Z, Taylor RJ, et al. Methadone and buprenorphine for the management of opioid dependence: a systematic review and economic evaluation. Health Technol Assess. 2007;11(9):1-171. https://doi.org/ 10.3310/hta11090 PMID: 17313907.
29. Xiao-Feng G. Health technology assessment of two antituberculosis regimens and a correspondence analysis between tuberculosis inpatient attributes and treatment results obtained in general hospitals of China.scholarly journal. [Doctoral Dissertation]. Sichuan University.
30. Gumbs PD, Verschuren MW, Mantel-Teeuwisse AK, de Wit AG, de Boer A, Klungel OH. Economic evaluations of cholesterol-lowering drugs: a critical and systematic review. Pharmacoeconomics. 2007;25(3):187-199. https://doi.org/ 10.2165/00019053-200725030-00002 PMID: 17335305.
31. Jin-hui T. Health technology assessment of antithymocyte globulin and basiliximab based on the new triple immunosuppression after kidney transplantation.scholarly journal. [Master Thesis]. Lanzhou University.
32. Li H, Banerjee S, Dunfield L, Kirby J, Jones M, Hamilton J, et al. Recombinant human growth hormone for treatment of Turner Syndrome: systematic review and economic evaluation. Health Technology Assessment Database. 2007;(96):1-56.
33. McLeod C, Bagust A, Boland A, Dagenais P, Dickson R, Dundar Y, et al. Adalimumab, etanercept and infliximab for the treatment of ankylosing spondylitis: a systematic review and economic evaluation. Health Technol Assess. 2007;11(28):1-158. https://doi.org/ 10.3310/hta11280 PMID: DARE-12008102719
34. Soares-Weiser K, Bravo Vergel Y, Beynon S, Dunn G, Barbieri M, Duffy S, et al. A systematic review and economic model of the clinical effectiveness and cost-effectiveness of interventions for preventing relapse in people with bipolar disorder. Health Technol Assess. 2007;11(39):1-206. https://doi.org/ 10.3310/hta11390 PMID: 17903393.
35. Sun X, Qin WX, Li YP, Jiang XH. Comparative cost-effectiveness of antiviral therapies in patients with chronic hepatitis B: a systematic review of economic evidence. J Gastroenterol Hepatol. 2007;22(9):1369-1377. https://doi.org/ 10.1111/j.1440-1746.2007.05068.x PMID: 17716343.
36. Ward S, Lloyd Jones M, Pandor A, Holmes M, Ara R, Ryan A, et al. A systematic review and economic evaluation of statins for the prevention of coronary events. Health Technol Assess. 2007;11(14):1-160. https://doi.org/ 10.1108/cgij.2007.24812dae.001 PMID: 17408535.
37. Wen-yuan L. Economical Assessment of Torasemide Versus Furosemide for Chronic Heart Failure.scholarly journal. [Master Thesis]. Lanzhou University.
38. Anonychuk AM, Tricco AC, Bauch CT, Pham B, Gilca V, Duval B, et al. Cost-effectiveness analyses of hepatitis A vaccine: A systematic review to explore the effect of methodological quality on the economic attractiveness of vaccination strategies. Pharmacoeconomics. 2008;26(1):17-32. https://doi.org/ 1170-7690/08/0001-0017/$48.00/0.
39. Chen M, Zhang LL, Hu M, Gao J, Tong RS. Cost-effectiveness of treatment for acute childhood idiopathic thrombocytopenic purpura (ITP)-a systematic review. J Int Med Res. 2008;36(3):572-578. https://doi.org/ 10.1177/147323000803600324 PMID: 18534141.
40. Cranny G, Elliott R, Weatherly H, Chambers D, Hawkins N, Myers L, et al. A systematic review and economic model of switching from non-glycopeptide to glycopeptide antibiotic prophylaxis for surgery. Health Technol Assess. 2008;12(1): 1-147. https://doi.org/ 10.3310/hta12010 PMID: 18093447.
41. Shepherd J, Rogers G, Anderson R, Main C, Thompson-Coon J, Hartwell D, et al. Systematic review and economic analysis of the comparative effectiveness of different inhaled corticosteroids and their usage with long-acting beta2 agonists for the treatment of chronic asthma in adults and children aged 12 years and over. Health Technol Assess. 2008;12(19):1-360. https://doi.org/ 10.1093/heapol/czn008 PMID: 18485271.
42. Wang D, Cummins C, Bayliss S, Sandercock J, Burls A. Immunoprophylaxis against respiratory syncytial virus (RSV) with palivizumab in children: a systematic review and economic evaluation. Health Technol Assess. 2008;12(36):1-86. https://doi.org/ 10.3310/hta12360 PMID: 19049692.
43. Burch J, Paulden M, Conti S, Stock C, Corbett M, Welton NJ, et al. Antiviral drugs for the treatment of influenza: a systematic review and economic evaluation. Health Technol Assess. 2009;13(58):1-265. https://doi.org/ 10.3310/hta13580 PMID: 19954682.
44. Chen YF, Jowett S, Barton P, Malottki K, Hyde C, Gibbs JS, et al. Clinical and cost-effectiveness of epoprostenol, iloprost, bosentan, sitaxentan and sildenafil for pulmonary arterial hypertension within their licensed indications: a systematic review and economic evaluation. Health Technol Assess. 2009;13(49):1-320. https://doi.org/ 10.3310/hta13490 PMID: 19863849.
45. Griffiths UK, Miners A. Economic evaluations of Haemophilus influenzae type b vaccine: Systematic review of the literature. Expert Review of Pharmacoeconomics and Outcomes Research. 2009;9(4):333-346. https://doi.org/ 10.1586/erp.09.38.
46. Jeurissen S, Makar A. Epidemiological and economic impact of human papillomavirus vaccines. Int J Gynecol Cancer. 2009;19(4):761-771. https://doi.org/ 10.1111/IGC.0b013e3181a130c0 PMID: 19509585.
47. Knight C, Dano AM, Kennedy-Martin T. A systematic review of the cost-effectiveness of rFVIIa and APCC in the treatment of minor/moderate bleeding episodes for haemophilia patients with inhibitors. Haemophilia. 2009;15(2):405-419. https://doi.org/ 10.1111/j.1365-2516.2008.01969.x PMID: 19187191.
48. LAN Ying LR, ZHANG Ling-li. Pharmacoeconomics of Methylphenidate for Attention Deficit Hyperactivity Disorder in Children :A Systematic Evaluation. C hina Pharmacy. 2009;(23). https://doi.org/ 10.1109/JSTARS.2010.2060473.
49. Pohar SL, Tsakonas E, Murphy G, Anderson D, Carney D, Moltzan C, et al. Recombinant activated Factor VII in treatment of hemorrhage unrelated to hemophilia: a systematic review and economic evaluation (Structured abstract). Health Technology Assessment Database. 2009;(118):1-168.
50. Simoens S, Laekeman G. Pharmacotherapy of allergic rhinitis: A pharmaco-economic approach. Allergy: European Journal of Allergy and Clinical Immunology. 2009;64(1):85-95. https://doi.org/ 10.1111/j.1398-9995.2008.01909.x .
51. Tonelli M, Lloyd A, Lee H, Wiebe N, Hemmelgarn B, Reiman T, et al. Erythropoiesis-stimulating agents for anemia of cancer or of chemotherapy: systematic review and economic evaluation. Database of Abstracts of Reviews of Effects. 2009;(2):1-187. https://doi.org/ PMID: DARE-12010001605
52. Bahadori K, Quon BS, Doyle-Waters MM, Marra C, Fitzgerald JM. A systematic review of economic evaluations of therapy in asthma. J Asthma Allergy. 2010;3:33-42. https://doi.org/ 10.2147/JAA.S11038 PMID: 21437038.
53. Kapoor A, Chuang W, Radhakrishnan N, Smith KJ, Berlowitz D, Segal JB, et al. Cost effectiveness of venous thromboembolism pharmacological prophylaxis in total hip and knee replacement: A systematic review. Pharmacoeconomics. 2010;28(7):521-538. https://doi.org/ 10.2165/11535210-000000000-00000 .
54. McKenna C, Burch J, Suekarran S, Walker S, Bakhai A, Witte K, et al. A systematic review and economic evaluation of the clinical effectiveness and cost-effectiveness of aldosterone antagonists for postmyocardial infarction heart failure. Health Technol Assess. 2010;14(24):1-162. https://doi.org/ 10.3310/hta14240 PMID: 20492762.
55. Murphy G, Tsakonas E, Ndegwa S, Anderson D, Barkun J, Mierzwinski-Urban M. Recombinant activated factor VII for prevention of bleeding unrelated to hemophilia: clinical and economic systematic review. Health Technology Assessment Database. 2010;(2):1-114.
56. Takeda A, Cooper K, Bird A, Baxter L, Frampton GK, Gospodarevskaya E, et al. Recombinant human growth hormone for the treatment of growth disorders in children: A systematic review and economic evaluation. Health Technology Assessment. 2010;14(42):1-237. https://doi.org/ 10.3310/hta14420.
57. Valentine WJ, Pollock RF, Plun-Favreau J, White J. Systematic review of the cost-effectiveness of biphasic insulin aspart 30 in type 2 diabetes. Curr Med Res Opin. 2010;26(6):1399-1412. https://doi.org/ 10.1185/03007991003689381 PMID: 20387997.
58. Boonacker CWB, Broos PH, Sanders EAM, Schilder AGM, Rovers MM. Cost effectiveness of pneumococcal conjugate vaccination against acute otitis media in children: A review. Pharmacoeconomics. 2011;29(3):199-211. https://doi.org/ 1170-7690/11/0003-0199/$49.95/0.
59. Dretzke J, Edlin R, Round J, Connock M, Hulme C, Czeczot J, et al. A systematic review and economic evaluation of the use of tumour necrosis factor-alpha (TNF-alpha) inhibitors, adalimumab and infliximab, for Crohn's disease. Health Technol Assess. 2011;15(6):1-244. https://doi.org/ 10.3310/hta15060 PMID: 21291629.
60. Hartwell D, Jones J, Baxter L, Shepherd J. Peginterferon alfa and ribavirin for chronic hepatitis c in patients eligible for shortened treatment, re-treatment or in HCV/HIV co-infection: A systematic review and economic evaluation. Health Technology Assessment. 2011;15(17):1-208. https://doi.org/ 10.3310/hta15170 .
61. Hislop J, Quayyum Z, Elders A, Fraser C, Jenkinson D, Mowatt G, et al. Clinical effectiveness and cost-effectiveness of imatinib dose escalation for the treatment of unresectable and/or metastatic gastrointestinal stromal tumours that have progressed on treatment at a dose of 400 mg/day: a systematic review and economic evaluation. Health Technol Assess. 2011;15(25):1-178. https://doi.org/ 10.3310/hta15250 PMID: 21689502.
62. Malottki K, Barton P, Tsourapas A, Uthman AO, Liu Z, Routh K, et al. Adalimumab, etanercept, infliximab, rituximab and abatacept for the treatment of rheumatoid arthritis after the failure of a TNF inhibitor: a systematic review and economic evaluation. Health Technology Assessment Database. 2011;15(14):1-301. https://doi.org/ 10.3310/hta15140.
63. Schwappach D, Popova S, Mohapatra S, Patra J, Godinho A, Rehm J. Strategies for evaluating the economic value of drugs in alcohol dependence treatment. Drug and Alcohol Dependence. 2011;122(3):165-173. https://doi.org/ 10.1016/j.drugalcdep.2011.08.026.
64. Squires H, Simpson E, Meng Y, Harnan S, Stevens J, Wong R, et al. A systematic review and economic evaluation of cilostazol, naftidrofuryl oxalate, pentoxifylline and inositol nicotinate for the treatment of intermittent claudication in people with peripheral arterial disease. Health technology assessment (Winchester, England). 2011;15(40):1-210. https://doi.org/ 10.3310/hta15400 .
65. Tsimicalis A, Stevens B, Ungar WJ, McKeever P, Greenberg M. The cost of childhood cancer from the family's perspective: A critical review. Pediatric Blood and Cancer. 2011;56(5):707-717. https://doi.org/ 10.1002/pbc.22685.
66. Auweiler PW, Muller D, Stock S, Gerber A. Cost effectiveness of rituximab for non-Hodgkin's lymphoma: a systematic review. Pharmacoeconomics. 2012;30(7):537-549. https://doi.org/ 10.2165/11591160-000000000-00000 PMID: 22612993.
67. Bongers ML, Coupe VM, Jansma EP, Smit EF, Uyl-de Groot CA. Cost effectiveness of treatment with new agents in advanced non-small-cell lung cancer: a systematic review. Pharmacoeconomics. 2012;30(1):17-34. https://doi.org/ 10.2165/11595000-000000000-00000 PMID: 22201521.
68. De Waure C, Veneziano MA, Cadeddu C, Capizzi S, Specchia ML, Capri S, et al. Economic value of influenza vaccination. Human Vaccines and Immunotherapeutics. 2012;8(1):119-129. https://doi.org/ 10.4161/hv.8.1.18420 .
69. John-Baptiste A, Yeung MW, Leung V, van der Velde G, Krahn M. Cost effectiveness of hepatitis C-related interventions targeting substance users and other high-risk groups: a systematic review. Pharmacoeconomics. 2012;30(11):1015-1034. https://doi.org/ 10.2165/11597660-000000000-00000 PMID: 23050771.
70. Loveman E, Cooper K, Bryant J, Colquitt JL, Frampton GK, Clegg A. Dasatinib, high-dose imatinib and nilotinib for the treatment of imatinib-resistant chronic myeloid leukaemia: A systematic review and economic evaluation. Health Technology Assessment. 2012;16(23):1-137. https://doi.org/ 10.3310/hta16230 .
71. Muller D, Pulm J, Gandjour A. Cost-effectiveness of different strategies for selecting and treating individuals at increased risk of osteoporosis or osteopenia: a systematic review. Value Health. 2012;15(2):284-298. https://doi.org/ 10.1016/j.jval.2011.11.030 PMID: 22433760.
72. Papaioannou D, Rafia R, Rathbone J, Stevenson M, Buckley Woods H, Stevens J. Rituximab for the first-line treatment of stage III-IV follicular lymphoma (review of Technology Appraisal No. 110): a systematic review and economic evaluation. Health Technol Assess. 2012;16(37):1-253. https://doi.org/ 10.3310/hta16370 PMID: 23021127.
73. Seto K, Marra F, Raymakers A, Marra CA. The cost effectiveness of human papillomavirus vaccines: a systematic review. Drugs. 2012;72(5):715-743. https://doi.org/ 10.2165/11599470-000000000-00000 PMID: 22413761.
74. Ying HQL. Pharmacoeconomics of 5-HT3 Serotonin Receptor Antagonist in the Prevention of Chemotherapeutic Drug Induced Vomiting in China: a Systematic Evaluation. China Pharmacy. 2012;(22):2020-2023.
75. Achilla E, McCrone P. The cost effectiveness of long-acting/extended-release antipsychotics for the treatment of schizophrenia: A systematic review of economic evaluations. Applied health economics and health policy. 2013;11(2):95-106. https://doi.org/ 10.1007/s40258-013-0016-2 .
76. Athanasakis K, Petrakis I, Kyriopoulos J. Investigating the value of abatacept in the treatment of rheumatoid arthritis: a systematic review of cost-effectiveness studies. ISRN Rheumatol. 2013;2013:1-297. https://doi.org/ 10.1155/2013/256871 PMID: 23819062.
77. Babigumira JB, Morgan I, Levin A. Health economics of rubella: a systematic review to assess the value of rubella vaccination. BMC Public Health. 2013;13(of Publication: 2013):406-419. https://doi.org/ 10.1186/1471-2458-13-406.
78. Brown T, Pilkington G, Bagust A, Boland A, Oyee J, Tudur-Smith C, et al. Clinical effectiveness and cost-effectiveness of first-line chemotherapy for adult patients with locally advanced or metastatic non-small cell lung cancer: a systematic review and economic evaluation. Health technology assessment. 2013;17(31):1-278. https://doi.org/ 10.3310/hta17310.
79. Buti M, Oyaguez I, Lozano V, Casado MA. Cost effectiveness of first-line oral antiviral therapies for chronic hepatitis B : a systematic review. Pharmacoeconomics. 2013;31(1):63-75. https://doi.org/ 10.1007/s40273-012-0009-2 PMID: 23329593.
80. Hashemi-Meshkini A, Keshavarz K, Gharibnaseri Z, Kheirandish M, Kebriaeezadeh A, Nikfar S, et al. Cost-effectiveness analysis review of exemestane in the treatment of primary and advanced breast cancer. Arch Med Sci. 2013;9(3):472-478. https://doi.org/ 10.5114/aoms.2013.35347 PMID: 23847669.
81. Hiligsmann M, Boonen A, Dirksen CD, Ben Sedrine W, Reginster JY. Cost-effectiveness of denosumab in the treatment of postmenopausal osteoporotic women. Expert Rev Pharmacoecon Outcomes Res. 2013;13(1):19-28. https://doi.org/ 10.1586/erp.12.76 PMID: 23402442.
82. Hoyle M, Crathorne L, Peters J, Jones-Hughes T, Cooper C, Napier M, et al. The clinical effectiveness and cost-effectiveness of cetuximab (mono- or combination chemotherapy), bevacizumab (combination with non-oxaliplatin chemotherapy) and panitumumab (monotherapy) for the treatment of metastatic colorectal cancer after first-line chemotherapy (review of technology appraisal no. 150 and part review of technology appraisal no. 118): A systematic review and economic model. Health Technology Assessment. 2013;17(14):1-144. https://doi.org/ 10.3310/HTA17140.
83. Marshall S, Fearon P, Dawson J, Quinn TJ. Stop the clots, but at what cost? Pharmacoeconomics of dabigatran etexilate for the prevention of stroke in subjects with atrial fibrillation: a systematic literature review. Expert Rev Pharmacoecon Outcomes Res. 2013;13(1):29-42. https://doi.org/ 10.1586/erp.12.79 PMID: 23402443.
84. Meadows A, Kaambwa B, Novielli N, Huissoon A, Fry-Smith A, Meads C, et al. A systematic review and economic evaluation of subcutaneous and sublingual allergen immunotherapy in adults and children with seasonal allergic rhinitis. Health Technology Assessment. 2013;17(27):1-340. https://doi.org/ 10.3310/hta17270.
85. Milte RK, Ratcliffe J, Miller MD, Crotty M. Economic evaluation for protein and energy supplementation in adults: Opportunities to strengthen the evidence. European Journal of Clinical Nutrition. 2013;67(12):1243-1250. https://doi.org/ 10.1038/ejcn.2013.206.
86. Norman G, Faria R, Paton F, Llewellyn A, Fox D, Palmer S, et al. Omalizumab for the treatment of severe persistent allergic asthma: a systematic review and economic evaluation. Health technology assessment (Winchester, England). 2013;17(52): 1-342. https://doi.org/ 10.3310/hta17520.
87. Sanz-Granda A, Hidalgo A, del Llano JE, Rovira J. Analysis of economic evaluations of pharmacological cancer treatments in Spain between 1990 and 2010. Clin Transl Oncol. 2013;15(1):9-19. https://doi.org/ 10.1007/s12094-012-0934-8 PMID: 23180344.
88. Simoens S. Cost-effectiveness of pharmacotherapy for COPD in ambulatory care: a review (Provisional abstract). Journal of Evaluation in Clinical Practice. 2013;(6):1004-1011. https://doi.org/ 10.1111/jep.12034 PMID: DARE-12014001429
89. Sun Yan-kun GLL, Li You-ping. Pharmacoeconomic Evaluation on Chemotherapy Combined with Rituximab for Non-Hodgkin's Lymphoma: A Systematic Review. Chin J Evid-based Med. 2013;13(6):700-708. https://doi.org/ 10.7507/1672-2531.20130125.
90. Szucs TD, Pfeil AM. A systematic review of the cost effectiveness of herpes zoster vaccination. Pharmacoeconomics. 2013;31(2):125-136. https://doi.org/ 10.1007/s40273-012-0020-7.
91. Unim B, Saulle R, Boccalini S, Taddei C, Ceccherini V, Boccia A, et al. Economic evaluation of varicella vaccination : Results of a systematic review. Human Vaccines and Immunotherapeutics. 2013;9(9):1932-1942. https://doi.org/ 10.4161/hv.25228.
92. Broder MS, Faria C, Powers A, Sunderji J, Cherepanov D. The impact of 5-HT3RA use on cost and utilization in patients with chemotherapy-induced nausea and vomiting: Systematic review of the literature. American Health and Drug Benefits. 2014;7(3):171-182. https://doi.org/ 10.1109/TENCON.1990.152691.
93. Fang Yu HM. Pharmacoeconomic of GLP-1 Receptor Agonist vervus DPP-4 Inhibitor for treating diabetes： A Systematic Evaluation. 2014 China Pharmaceutical Conference and the 14th China Pharmacist Week Proceedings. 2014:1-14.
94. Freijer K, Bours MJ, Nuijten MJ, Poley MJ, Meijers JM, Halfens RJ, et al. The economic value of enteral medical nutrition in the management of disease-related malnutrition: a systematic review. J Am Med Dir Assoc. 2014;15(1):17-29. https://doi.org/ 10.1016/j.jamda.2013.09.005 PMID: 24239013.
95. Gialama F, Miloni E, Maniadakis N. Cost Effectiveness of Treatments for non-ST-segment elevation Acute Coronary Syndrome. Pharmacoeconomics. 2014;32(11):1063-1078. https://doi.org/ 10.1007/s40273-014-0191-5.
96. Huang Y, Zhou Q, Haaijer-Ruskamp FM, Postma MJ. Economic evaluations of angiotensin-converting enzyme inhibitors and angiotensin II receptor blockers in type 2 diabetic nephropathy: a systematic review. BMC Nephrol. 2014;15(15):1-17. https://doi.org/ 10.1186/1471-2369-15-15 PMID: 24428868.
97. Joensuu JT, Aaltonen KJ, Huoponen S, Konttinen YT, Nordstrom D, Blom M. The cost-effectiveness of biologis for the treatment of rheumatoid arthritis: A systematic review. Annals of the Rheumatic Diseases. 2014;10(3):e0119683. https://doi.org/ 10.1371/journal.pone.0119683.
98. Kawai K, Preaud E, Baron-Papillon F, Largeron N, Acosta CJ. Cost-effectiveness of vaccination against herpes zoster and postherpetic neuralgia: a critical review. vaccine. 2014;32(2014):1645-1653. https://doi.org/ 10.1016/j.jval.2013.08.070 PMID: DARE-12014015124.
99. Lange A, Prenzler A, Frank M, Kirstein M, Vogel A, von der Schulenburg JM. A systematic review of cost-effectiveness of monoclonal antibodies for metastatic colorectal cancer. Eur J Cancer. 2014;50(1):40-49. https://doi.org/ 10.1016/j.ejca.2013.08.008 PMID: 24011538.
100. Li Qian MA. Pharmacoeconomic Evaluation on Chemotherapy Combined with Rituximab for the Treatment of NonHodgkin's Lymphoma. PROGRESS IN PHARMACEUTICAL SCIENCES. 2014;38(05):362-369.
101. Rivero-Santana A, Cuellar-Pompa L, Sanchez-Gomez LM, Perestelo-Perez L, Serrano-Aguilar P. Effectiveness and cost-effectiveness of different immunization strategies against whooping cough to reduce child morbidity and mortality. Health Policy. 2014;115(1):82-91. https://doi.org/ 10.1016/j.healthpol.2013.12.007 PMID: 24444703.
102. Tricco AC, Ashoor HM, Antony J, Beyene J, Veroniki AA, Isaranuwatchai W, et al. Safety, effectiveness, and cost effectiveness of long acting versus intermediate acting insulin for patients with type 1 diabetes: systematic review and network meta-analysis. BMJ. 2014;349:g5459. https://doi.org/ 10.1136/bmj.g5459 PMID: 25274009.
103. Zhang W, Islam N, Ma C, Anis AH. Systematic review of cost-effectiveness analyses of treatments for psoriasis (Provisional abstract). PharmacoEconomics. 2014;33(4):327-340. https://doi.org/ 10.1007/s40273-014-0244-9 PMID: DARE-12014071027.
104. Geng J, Yu H, Mao Y, Zhang P, Chen Y. Cost Effectiveness of Dipeptidyl Peptidase-4 Inhibitors for Type 2 Diabetes. Pharmacoeconomics. 2015;33(6):581-597. https://doi.org/ 10.1007/s40273-015-0266-y.
105. Greenhalgh J, Bagust A, Boland A, Dwan K, Beale S, Hockenhull J, et al. Erlotinib and gefitinib for treating non-small cell lung cancer that has progressed following prior chemotherapy (review of NICE technology appraisals 162 and 175): a systematic review and economic evaluation. Health Technol Assess. 2015;19(47):1-134. https://doi.org/ 10.3310/hta19470 PMID: 26134145.
106. Hiligsmann M, Evers SM, Ben Sedrine W, Kanis JA, Ramaekers B, Reginster JY, et al. A systematic review of cost-effectiveness analyses of drugs for postmenopausal osteoporosis. Pharmacoeconomics. 2015;33(3):205-224. https://doi.org/ 10.1007/s40273-014-0231-1 PMID: 25377850.
107. Peng Yan-qin YZ, Wang Guo-dong. A systematic review of the pharmacoeconomics research of the 23-valent pneumococcal polysaccharide vaccine for the elderly. China Pharmacy. 2015;26(36):5116-5118.
108. Poonawalla IB, Parikh RC, Du XL, VonVille HM, Lairson DR. Cost effectiveness of chemotherapeutic agents and targeted biologics in ovarian cancer: a systematic review. Pharmacoeconomics. 2015;33(11):1155-1185. https://doi.org/ 10.1007/s40273-015-0304-9.
109. Roze S, Smith‐Palmer J, Valentine W, De Portu S, Nørgaard K, Pickup J. Cost‐effectiveness of continuous subcutaneous insulin infusion versus multiple daily injections of insulin in Type 1 diabetes: a systematic review. Diabetic Medicine. 2015;32(11):1415-1424. https://doi.org/ 10.1111/dme.12792.
110. Wang Hai-yin HJ-j, Lin Xia. A systematic review of the pharmacoeconomics of budesonide/formoterol versus fluticasone/salmeterol in the treatment of asthma. China Pharmacy. 2015;26(18):2527-2529.
111. Yahia M-BBH, Jouin-Bortolotti A, Dervaux B. Extending the human papillomavirus vaccination programme to include males in high-income countries: a systematic review of the cost-effectiveness studies. Clinical drug investigation. 2015;35(8):471-485. https://doi.org/ 10.1007/s40261-015-0308-4 .
112. Ahmadiani S, Nikfar S, Karimi S, Jamshidi AR, Akbari-Sari A, Kebriaeezadeh A. Rituximab as first choice for patients with refractory rheumatoid arthritis: cost-effectiveness analysis in Iran based on a systematic review and meta-analysis. Rheumatology international. 2016;36(9):1291-1300. https://doi.org/ 10.1007/s00296-016-3484-5 .
113. Archer R, Tappenden P, Ren S, James M-S, Harvey R, Basarir H, et al. Infliximab, adalimumab and golimumab for treating moderately to severely active ulcerative colitis after the failure of conventional therapy (including a review of TA140 and TA262): clinical effectiveness systematic review and economic model. Health Technoogyl Assessment. 2016;20:No.6. https://doi.org/ 10.3310/hta20390 .
114. Chit A, Lee JK, Shim M, Nguyen VH, Grootendorst P, Wu J, et al. Economic evaluation of vaccines in Canada: A systematic review. Human vaccines & immunotherapeutics. 2016;12(5):1257-1264. https://doi.org/ 10.1080/21645515.2015.1137405.
115. Jones-Hughes T, Snowsill T, Haasova M, Coelho H, Crathorne L, Cooper C, et al. Immunosuppressive therapy for kidney transplantation in adults: a systematic review and economic model. Health Technology Assessment. 2016;20(62):1-594. https://doi.org/ 10.3310/hta20620 .
116. Kourlaba G, Gialama F, Tsioufis K, Maniadakis N. A literature review to evaluate the clinical and economic value of olmesartan for the treatment of hypertensive patients. International journal of cardiology. 2016;221:60-74.
117. Liberato NL, Marchetti M. Cost-effectiveness of non-vitamin K antagonist oral anticoagulants for stroke prevention in non-valvular atrial fibrillation: a systematic and qualitative review. Expert review of pharmacoeconomics & outcomes research. 2016;16(2):221-235. https://doi.org/ 10.1586/14737167.2016.1147351 .
118. Men Peng ZJ-w, Tang Hui-lin, Zhai Suo-di. A systematic review of pharmacoeconomics of saxagliptin in the treatment of type 2 diabetes. Chinese Pharmaceutical Journal. 2016;51(12):1044-1048.
119. Mistro S, Rosa L, Gomes B, Miranda L, Badaró R. Cost-effectiveness of caspofungin versus liposomal amphotericin B in the treatment of systemic fungal infections: a systematic review of economic analyses. Expert review of pharmacoeconomics & outcomes research. 2016;16(4):465-473. https://doi.org/ 10.1080/14737167.2016.1202766.
120. Nerich V, Saing S, Gamper EM, Kemmler G, Daval F, Pivot X, et al. Cost–utility analyses of drug therapies in breast cancer: a systematic review. Breast cancer research and treatment. 2016;159(3):407-424. https://doi.org/ 10.1007/s10549-016-3924-7.
121. Vellopoulou K, Kourlaba G, Maniadakis N, Vardas P. A literature review to evaluate the economic value of ranolazine for the symptomatic treatment of chronic angina pectoris. International journal of cardiology. 2016;211:105-111. https://doi.org/ 10.1016/j.ijcard.2016.02.140.
122. Andronis L, Goranitis I, Bayliss S, Duarte R. Cost-Effectiveness of Treatments for the Management of Bone Metastases: A Systematic Literature Review. Pharmacoeconomics. 2017;36(3):301-322. https://doi.org/ 10.1007/s40273-017-0595-0 PMID: 29224174.
123. Chhatwal J, He T, Hur C, Lopez-Olivo MA. Direct-acting antiviral agents for patients with hepatitis C virus genotype 1 infection are cost-saving. Clinical Gastroenterology and Hepatology. 2017;15(6):827-837. https://doi.org/ 10.1016/j.cgh.2016.09.015.
124. Chongmelaxme B, Hammanee M, Phooaphirak W, Kotirum S, Hutubessy R, Chaiyakunapruk N. Economic evaluations of Haemophilus influenzae type b (Hib) vaccine: a systematic review. Journal of medical economics. 2017;20(10):1094-1106. https://doi.org/ 10.1080/13696998.2017.1359181.
125. Corbett M, Chehadah F, Biswas M, Moe-Byrne T, Palmer S, Soares M, et al. Certolizumab pegol and secukinumab for treating active psoriatic arthritis following inadequate response to disease-modifying antirheumatic drugs: a systematic review and economic evaluation. Health Technology Assessment. 2017:1-326. https://doi.org/ 10.3310/hta21560 .
126. de Boer PT, van Maanen BM, Damm O, Ultsch B, Dolk FC, Crépey P, et al. A systematic review of the health economic consequences of quadrivalent influenza vaccination. Expert review of pharmacoeconomics & outcomes research. 2017;17(3):249-265. https://doi.org/ 10.1080/14737167.2017.1343145 .
127. Didik S. The health-economic studies of HPV vaccination in Southeast Asian countries: a systematic review. REPORT OF THE PLAGIARISM CHECK. 2017:1-22. https://doi.org/ 10.1080/14760584.2017.1357472 .
128. Ding Haiying TY, Xin Wenxiu, Huang Ping. A Literature Review on Economic Evaluation of Bevacizumab in Treatment of Ovarian Cancer. Chin J Mod Appl Pharm. 2017;34(11):1621-1627.
129. Herzog S, Shanahan M, Grimison P, Tran A, Wong N, Lintzeris N, et al. Systematic Review of the Costs and Benefits of Prescribed Cannabis-Based Medicines for the Management of Chronic Illness: Lessons from Multiple Sclerosis. Pharmacoeconomics. 2017;36(24):1-12. https://doi.org/ 10.1007/s40273-017-0565-6 PMID: 28866778.
130. Iannazzo S, Iliza AC, Perrault L. Disease-Modifying Therapies for Multiple Sclerosis: A Systematic Literature Review of Cost-Effectiveness Studies. Pharmacoeconomics. 2017;36(2):189-204. https://doi.org/ 10.1007/s40273-017-0577-2 PMID: 29032493.
131. Johnston R, Uthman O, Cummins E, Clar C, Royle P, Colquitt J, et al. Canagliflozin, dapagliflozin and empagliflozin monotherapy for treating type 2 diabetes: systematic review and economic evaluation. Health Technology Assessment. 2017;21(2):1-218. https://doi.org/ 10.3310/hta21020 .
132. Kotirum S, Vutipongsatorn N, Kongpakwattana K, Hutubessy R, Chaiyakunapruk N. Global economic evaluations of rotavirus vaccines: A systematic review. Vaccine. 2017;35(26):3364-3386. https://doi.org/ 10.1016/j.vaccine.2017.04.051.
133. Le LK, Hay P, Mihalopoulos C. A systematic review of cost-effectiveness studies of prevention and treatment for eating disorders. Aust N Z J Psychiatry. 2017;52(4):328-338. https://doi.org/ 10.1177/0004867417739690 PMID: 29113456.
134. Melendez-Torres G, Auguste P, Armoiry X, Maheswaran H, Madan J, Kan A, et al. Clinical effectiveness and cost-effectiveness of beta-interferon and glatiramer acetate for treating multiple sclerosis: systematic review and economic evaluation. Health Technology Assessment. 2017;21(52):1-352. https://doi.org/ 10.3310/hta21520 .
135. Wijnen BF, van Mastrigt GA, Evers SM, Gershuni O, Lambrechts DA, Majoie MH, et al. A systematic review of economic evaluations of treatments for patients with epilepsy. Epilepsia. 2017;58(5):706-726. https://doi.org/ 10.1111/epi.13655 .
136. Al Kadour A, Al Marridi W, Al-Badriyeh D. Pharmacoeconomics evaluations of oral anticancer agents: systematic review of characteristics, methodological trends, and reporting quality. Value in health regional issues. 2018;16:46-60. https://doi.org/ 10.1016/j.vhri.2018.05.003.
137. Camacho EM, Shields GE. Cost-effectiveness of interventions for perinatal anxiety and/or depression: a systematic review. BMJ Open. 2018;8(8):e022022. https://doi.org/ 10.1136/bmjopen-2018-022022 PMID: 30099399.
138. Castro R, Crathorne L, Perazzo H, Silva J, Cooper C, Varley-Campbell J, et al. Cost-effectiveness of diagnostic and therapeutic interventions for chronic hepatitis C: a systematic review of model-based analyses. BMC Medical Research Methodology. 2018;18(1):53-69. https://doi.org/ 10.1186/s12874-018-0515-9 PMID: 29895281.
139. D'Angiolella LS, Cortesi PA, Lafranconi A, Micale M, Mangano S, Cesana G, et al. Cost and Cost Effectiveness of Treatments for Psoriatic Arthritis: A Systematic Literature Review. Pharmacoeconomics. 2018;36(5):567-589. https://doi.org/ 10.1007/s40273-018-0618-5 PMID: 29441473.
140. Ebrahem AS, Oremus M. A pharmacoeconomic evaluation of cholinesterase inhibitors and memantine for the treatment of Alzheimer’s disease. Expert opinion on pharmacotherapy. 2018;19(11):1245-1259. https://doi.org/ 10.1080/14656566.2018.1499727.
141. Elshout M, Webers CAB, van der Reis MI, Schouten J. A systematic review on the quality, validity and usefulness of current cost-effectiveness studies for treatments of neovascular age-related macular degeneration. Acta Ophthalmol (Oxf). 2018;96(8):770-778. https://doi.org/ 10.1111/aos.13824 PMID: 29862641.
142. Gedge LM, Bettis AA, Bradley MH, Hollingsworth TD, Turner HC. Economic evaluations of lymphatic filariasis interventions: a systematic review and research needs. Parasit Vectors. 2018;11(1):75-93. https://doi.org/ 10.1186/s13071-018-2616-z PMID: 29391042.
143. Grochtdreis T, Konig HH, Dobruschkin A, von Amsberg G, Dams J. Cost-effectiveness analyses and cost analyses in castration-resistant prostate cancer: A systematic review. PLoS ONE. 2018;13(12):e0208063. https://doi.org/ 10.1371/journal.pone.0208063 PMID: 30517165.
144. Han D, Iragorri N, Clement F, Lorenzetti D, Spackman E. Cost Effectiveness of Treatments for Chronic Constipation: A Systematic Review. Pharmacoeconomics. 2018;36(4):435-449. https://doi.org/ 10.1007/s40273-018-0609-6 PMID: 29352437.
145. Hsiao A, Hall AH, Mogasale V, Quentin W. The health economics of cholera: A systematic review. Vaccine. 2018;36(30):4404-4424. https://doi.org/ 10.1016/j.vaccine.2018.05.120 PMID: 29907482.
146. Hui C, Dunn J, Morton R, Staub LP, Tran A, Hargreaves S, et al. Interventions to Improve Vaccination Uptake and Cost Effectiveness of Vaccination Strategies in Newly Arrived Migrants in the EU/EEA: A Systematic Review. International Journal of Environmental Research & Public Health. 2018;15(15):2065-2078. https://doi.org/ 10.3390/ijerph15102065 PMID: 30241320.
147. Jafari A, Rezapour A, Hajahmadi M. Cost-effectiveness of B-type natriuretic peptide-guided care in patients with heart failure: a systematic review. Heart Fail Rev. 2018;23(5):693-700. https://doi.org/ 10.1007/s10741-018-9710-3 PMID: 29744629.
148. Jean L, Audrey M, Beauchemin C, Consortium O. Economic Evaluations of Treatments for Inflammatory Bowel Diseases: A Literature Review. Can J Gastroenterol Hepatol. 2018;2018:No.7439730. https://doi.org/ 10.1155/2018/7439730 PMID: 30009158.
149. Kromer C, Celis D, Sonntag D, Peitsch WK. Biologicals and small molecules in psoriasis: A systematic review of economic evaluations. PLoS ONE. 2018;13(1):e0189765. https://doi.org/ 10.1371/journal.pone.0189765 PMID: 29298315.
150. Le P, Nghiem VT, Mullen PD, Deshpande A. Cost-Effectiveness of Competing Treatment Strategies for Clostridium difficile Infection: A Systematic Review. Infect Control Hosp Epidemiol. 2018;39(4):412-424. https://doi.org/ 10.1017/ice.2017.303 PMID: 29463339.
151. Leonart LP, Borba HHL, Ferreira VL, Riveros BS, Pontarolo R. Cost-effectiveness of acromegaly treatments: a systematic review. Pituitary. 2018;21(6):642-652. https://doi.org/ 10.1007/s11102-018-0908-0 PMID: 30159696.
152. LI Chao LN, DONG Shu-jie, FENG Yu-fei. Systematic Review of Cost-effectiveness Analyses of Dabigatran for Anticoagulation Treatment in Atrial Fibrillation. Drug Evaluation. 2018;15(10):21-25.
153. McQueen RB, Sheehan DN, Whittington MD, van Boven JFM, Campbell JD. Cost-Effectiveness of Biological Asthma Treatments: A Systematic Review and Recommendations for Future Economic Evaluations. Pharmacoeconomics. 2018;36(8):957-971. https://doi.org/ 10.1007/s40273-018-0658-x PMID: 29736895.
154. Ng SS, Hutubessy R, Chaiyakunapruk N. Systematic review of cost-effectiveness studies of human papillomavirus (HPV) vaccination: 9-Valent vaccine, gender-neutral and multiple age cohort vaccination. Vaccine. 2018;36(19):2529-2544. https://doi.org/ 10.1016/j.vaccine.2018.03.024 PMID: 29625764.
155. Nishikawa AM, Sartori AMC, Mainardi GM, Freitas AC, Itria A, Novaes HMD, et al. Systematic review of economic evaluations of the 23-valent pneumococcal polysaccharide vaccine (PPV23) in individuals 60years of age or older. Vaccine. 2018;36(19):2510-2522. https://doi.org/ 10.1016/j.vaccine.2018.03.070 PMID: 29618414.
156. Petrou P. A systematic review of economic evaluations of tyrosine kinase inhibitors of vascular endothelial growth factor receptors, mammalian target of rapamycin inhibitors and programmed death-1 inhibitors in metastatic renal cell cancer. Expert Review of Pharmacoeconomics & Outcomes Research. 2018;18(3):255-265. https://doi.org/ 10.1080/14737167.2018.1439740 PMID: 29448845.
157. Puig-Junoy J, Pascual-Argente N, Puig-Codina L, Planellas L, Solozabal M. Cost-utility analysis of second-generation direct-acting antivirals for hepatitis C: a systematic review. Expert rev. 2018;12(12):1251-1263. https://doi.org/ 10.1080/17474124.2018.1540929 PMID: 30791790.
158. Rodriguez-Martinez CE, Sossa-Briceno MP, Castro-Rodriguez JA. Cost Effectiveness of Pharmacological Treatments for Asthma: A Systematic Review. Pharmacoeconomics. 2018;36(10):1165-1200. https://doi.org/ 10.1007/s40273-018-0668-8 PMID: 29869050.
159. Shih ST, Tonmukayakul U, Imms C, Reddihough D, Graham HK, Cox L, et al. Economic evaluation and cost of interventions for cerebral palsy: a systematic review. Developmental Medicine & Child Neurology. 2018;60(6):543-558. https://doi.org/ 10.1111/dmcn.13653 .
160. Silas OA, Achenbach CJ, Murphy RL, Hou L, Sagay SA, Banwat E, et al. Cost effectiveness of human papilloma virus vaccination in low and middle income countries: a systematic review of literature. Expert Rev Vaccines. 2018;17(1):91-98. https://doi.org/ 10.1080/14760584.2018.1411195 PMID: 29183182.
161. Teoh SL, Kotirum S, Hutubessy RCW, Chaiyakunapruk N. Global economic evaluation of oral cholera vaccine: A systematic review. Human vaccines & Immunotherapeutics. 2018;14(2):420-429. https://doi.org/ 10.1080/21645515.2017.1392422 PMID: 29099647.
162. Verma V, Sprave T, Haque W, Simone CB, 2nd, Chang JY, Welsh JW, et al. A systematic review of the cost and cost-effectiveness studies of immune checkpoint inhibitors. J Immunother Cancer. 2018;6(1):128. https://doi.org/ 10.1186/s40425-018-0442-7 PMID: 30470252.
163. Wortman MSH, Lokkerbol J, van der Wouden JC, Visser B, van der Horst HE, Olde Hartman TC. Cost-effectiveness of interventions for medically unexplained symptoms: A systematic review. PLoS ONE. 2018;13(10):e0205278. https://doi.org/ 10.1371/journal.pone.0205278 PMID: 30321193.
164. You R, Qian X, Tang W, Xie T, Zeng F, Chen J, et al. Cost Effectiveness of Bosentan for Pulmonary Arterial Hypertension: A Systematic Review. Can Respir J. 2018;2018:No.1015239. https://doi.org/ 10.1155/2018/1015239 PMID: 30581511.
165. Zhao Shuang QW-l, Qiu Zhixin, Li Lei, Li Wei-min. A systematic review of the cost-effectiveness of gefitinib for advanced non-small cell lung cancer patients. West China Medical Journal. 2018;33(1):1-8.
