# Supplementary material for: An overview of the characteristics and quality assessment criteria in systematic review of pharmacoeconomics
Source: PLoS One. 2021 Feb 8;16(2):e0246080. doi: 10.1371/journal.pone.0246080 (PMC7870091; doi:10.1371/journal.pone.0246080)
Supplement: S1 Table — (DOCX) [file pone.0246080.s003.docx]

**S1 Table. AMSTAR assessment scale for included 165 studies.**

| **No.** | **Study ID** | **Item1** | **Item 2** | **Item 3** | **Item 4** | **Item 5** | **Item 6** | **Item 7** | **Item 8** | **Item 9** | **Item 10** | **Scored** |
| --- | --- | --- | --- | --- | --- | --- | --- | --- | --- | --- | --- | --- |
| 1 | Lister-Sharp 2000 | Y | Y | Y | Y | Y | Y | Y | Y | C | Y | 9 |
| 2 | Jordon 2000 | Y | Y | Y | Y | Y | Y | Y | Y | C | Y | 9 |
| 3 | Forbes 2001 | Y | Y | Y | Y | Y | Y | Y | Y | C | Y | 9 |
| 4 | Meara 2001 | Y | Y | Y | Y | Y | Y | Y | Y | C | Y | 9 |
| 5 | Robinson 2002 | Y | Y | Y | Y | Y | Y | Y | Y | C | Y | 9 |
| 6 | Forbes 2002 | Y | Y | Y | Y | Y | Y | Y | Y | C | Y | 9 |
| 7 | Lewis 2002 | Y | Y | Y | Y | Y | Y | Y | Y | C | Y | 9 |
| 8 | Hancock 2002 | Y | Y | Y | Y | Y | Y | Y | Y | C | Y | 9 |
| 9 | Segal 2003 | N | Y | Y | Y | N | Y | Y | Y | C | N | 6 |
| 10 | Boland 2003 | Y | Y | Y | Y | Y | Y | Y | Y | C | Y | 9 |
| 11 | Hidalgo 2003 | Y | Y | Y | Y | Y | Y | Y | Y | C | Y | 9 |
| 12 | Dixon 2004 | N | C | Y | C | N | N | Y | N | C | Y | 3 |
| 13 | Brinsmead 2004 | N | C | Y | C | N | N | Y | N | C | Y | 3 |
| 14 | Main 2004 | Y | Y | Y | Y | Y | Y | Y | Y | C | Y | 9 |
| 15 | Jones 2004 | Y | Y | Y | Y | Y | Y | Y | Y | C | Y | 9 |
| 16 | Sun 2005 | N | C | Y | C | N | Y | Y | N | C | N | 3 |
| 17 | Xiong 2005 | N | Y | Y | Y | N | Y | Y | N | C | N | 5 |
| 18 | Wilby 2005 | Y | Y | Y | Y | Y | Y | Y | Y | C | Y | 9 |
| 19 | Sun 2006 | N | C | Y | Y | N | N | Y | N | C | N | 3 |
| 20 | Chen 2006 | Y | Y | Y | Y | Y | Y | Y | Y | C | Y | 9 |
| 21 | Woolacott 2006 | Y | Y | Y | Y | Y | Y | Y | Y | C | Y | 9 |
| 22 | Chen 2006 | Y | C | Y | Y | N | Y | Y | N | C | N | 5 |
| 23 | Sun 2007 | N | Y | Y | C | N | N | Y | N | C | N | 3 |
| 24 | Gumbs 2007 | N | C | Y | C | N | Y | Y | Y | C | Y | 5 |
| 25 | Li 2007 | N | Y | Y | Y | N | Y | Y | N | C | N | 5 |
| 26 | Tian 2007 | N | Y | Y | Y | N | Y | Y | N | C | N | 5 |
| 27 | Gao 2007 | N | Y | Y | Y | N | N | Y | Y | C | N | 5 |
| 28 | Soares-Weiser 2007 | Y | Y | Y | Y | Y | Y | Y | Y | C | Y | 9 |
| 29 | Collins 2007 | Y | Y | Y | Y | Y | Y | Y | Y | C | Y | 9 |
| 30 | Sun 2007 | Y | Y | Y | Y | Y | Y | Y | Y | C | Y | 9 |
| 31 | ConNck 2007 | Y | Y | Y | Y | Y | Y | Y | Y | C | Y | 9 |
| 32 | McLeod 2007 | Y | Y | Y | Y | Y | Y | Y | Y | C | Y | 9 |
| 33 | Huimin Li 2007 | Y | Y | Y | Y | N | Y | Y | Y | C | Y | 8 |
| 34 | ANnychuk 2008 | N | Y | Y | Y | N | Y | Y | N | C | Y | 6 |
| 35 | CHEN 2008 | N | C | Y | Y | N | Y | Y | Y | C | Y | 6 |
| 36 | Cranny 2008 | Y | Y | Y | Y | Y | Y | Y | Y | C | Y | 9 |
| 37 | Wang 2008 | Y | Y | Y | Y | Y | Y | Y | Y | C | Y | 9 |
| 38 | Knight 2009 | N | Y | Y | C | N | Y | Y | N | C | Y | 5 |
| 39 | Simoens 2009 | N | C | Y | C | N | N | Y | N | C | Y | 3 |
| 40 | Jeurissen 2009 | N | C | Y | C | N | N | Y | Y | C | N | 3 |
| 41 | Griffiths 2009 | N | C | Y | C | N | Y | Y | N | C | Y | 4 |
| 42 | Lan 2009 | N | C | Y | Y | N | N | Y | N | C | N | 3 |
| 43 | Pandor 2006 | Y | Y | Y | Y | Y | Y | Y | Y | C | Y | 9 |
| 44 | Chen 2009 | Y | Y | Y | Y | Y | Y | Y | Y | C | Y | 9 |
| 45 | Burch 2009 | Y | Y | Y | Y | Y | Y | Y | Y | C | Y | 9 |
| 46 | Sheri 2009 | Y | Y | Y | Y | Y | Y | Y | Y | C | Y | 9 |
| 47 | Tonelli 2009 | Y | Y | Y | Y | Y | Y | Y | Y | C | Y | 9 |
| 48 | McKenna 2010 | N | Y | Y | Y | Y | Y | Y | Y | C | Y | 8 |
| 49 | Murphy 2010 | N | Y | Y | Y | Y | Y | Y | Y | C | Y | 8 |
| 50 | Takeda 2010 | N | Y | Y | Y | Y | Y | Y | Y | C | C | 7 |
| 51 | Bahadori 2010 | N | Y | C | Y | N | Y | Y | N | C | Y | 5 |
| 52 | Kapoor 2010 | N | C | Y | C | N | Y | Y | N | C | N | 3 |
| 53 | Dretzke 2011 | N | Y | Y | Y | Y | Y | Y | Y | C | Y | 8 |
| 54 | Schwappach 2011 | N | C | Y | Y | N | Y | C | C | C | C | 3 |
| 55 | Tsimicalis 2011 | N | N | N | N | N | Y | Y | Y | C | C | 3 |
| 56 | Hartwell 2011 | N | Y | Y | Y | Y | Y | Y | Y | C | C | 7 |
| 57 | Hislop 2011 | N | Y | Y | C | Y | Y | Y | Y | C | C | 6 |
| 58 | Squires 2011 | N | Y | Y | Y | Y | Y | Y | Y | C | Y | 8 |
| 59 | Boonacker 2011 | N | Y | Y | C | N | Y | Y | Y | C | N | 5 |
| 60 | Auweiler 2012 | N | C | Y | Y | N | Y | Y | Y | C | C | 5 |
| 61 | Bongers 2012 | N | Y | Y | Y | N | Y | Y | Y | C | Y | 7 |
| 62 | Waure, 2012 | N | Y | N | Y | N | Y | Y | Y | C | N | 5 |
| 63 | John-Baptiste 2012 | N | Y | Y | Y | N | Y | Y | Y | C | Y | 7 |
| 64 | Müller 2012 | N | C | Y | C | N | Y | Y | Y | C | Y | 5 |
| 65 | Seto 2012 | N | C | Y | C | N | Y | C | C | C | C | 2 |
| 66 | He 2012 | N | N | N | N | N | Y | Y | Y | C | N | 3 |
| 67 | Loveman 2012 | Y | Y | Y | Y | Y | Y | Y | Y | C | Y | 9 |
| 68 | PapaioanNu 2012 | Y | Y | Y | Y | Y | Y | Y | Y | C | C | 8 |
| 69 | Achilla, E 2013 | N | C | Y | Y | N | Y | Y | Y | C | C | 5 |
| 70 | Athanasakis 2013 | N | C | Y | Y | N | Y | N | N | C | C | 3 |
| 71 | Babigumira 2013 | N | C | Y | Y | N | Y | N | N | C | C | 3 |
| 72 | Brown 2013 | N | Y | Y | Y | N | Y | Y | Y | Y | Y | 8 |
| 73 | Buti 2013 | N | C | Y | Y | N | Y | Y | Y | C | C | 5 |
| 74 | Hashemi-Meshkini 2013 | N | C | Y | C | N | Y | Y | Y | C | C | 4 |
| 75 | Hiligsmann 2013 | N | C | Y | Y | N | Y | Y | Y | C | Y | 6 |
| 76 | Marshall 2013 | N | C | Y | Y | N | Y | C | C | C | C | 3 |
| 77 | Milte 2013 | N | Y | Y | Y | N | Y | Y | Y | C | C | 6 |
| 78 | Sanz-Granda 2013 | N | C | Y | C | N | Y | Y | C | C | Y | 4 |
| 79 | Simoens 2013 | N | N | Y | C | N | N | C | C | C | N | 1 |
| 80 | Szucs 2013 | N | C | Y | Y | N | Y | N | N | C | Y | 4 |
| 81 | Unim 2013 | N | Y | Y | C | N | Y | Y | Y | C | C | 5 |
| 82 | Sun 2013 | N | N | Y | N | N | Y | Y | Y | C | N | 4 |
| 83 | Hoyle 2013 | Y | Y | Y | Y | N | Y | Y | Y | C | Y | 8 |
| 84 | Meadows 2013 | Y | Y | Y | Y | Y | Y | Y | Y | C | Y | 9 |
| 85 | Nrman 2013 | Y | Y | Y | Y | Y | Y | Y | Y | C | Y | 9 |
| 86 | Broder 2014 | N | C | Y | Y | N | Y | Y | Y | C | Y | 6 |
| 87 | Freijer BHS，2014 | N | Y | Y | Y | N | Y | Y | Y | C | N | 6 |
| 88 | Gialama 2014 | N | C | Y | Y | N | Y | Y | Y | C | Y | 6 |
| 89 | Huang 2014 | N | C | Y | Y | N | Y | C | C | C | C | 3 |
| 90 | Joensuu 2014 | N | Y | Y | Y | N | Y | Y | Y | C | C | 6 |
| 91 | Kawaia, 2014 | N | N | Y | Y | N | Y | Y | Y | C | C | 5 |
| 92 | Lange 2014 | N | Y | Y | Y | N | Y | Y | Y | C | C | 6 |
| 93 | Rivero-Santana 2014 | N | C | Y | Y | N | Y | Y | C | C | Y | 5 |
| 94 | Tricco 2014 | Y | Y | N | C | N | Y | Y | Y | C | C | 5 |
| 95 | Zhang 2014 | N | Y | Y | Y | N | Y | Y | Y | C | C | 6 |
| 96 | Fang 2014 | N | C | Y | N | N | Y | Y | Y | C | N | 4 |
| 97 | Li 2014 | N | N | Y | N | N | Y | Y | Y | C | N | 4 |
| 98 | Hiligsmann 2014 | N | C | Y | Y | N | Y | Y | Y | C | Y | 6 |
| 99 | Geng 2015 | N | C | Y | Y | N | Y | Y | Y | C | Y | 6 |
| 100 | Hiligsmann 2015 | N | C | Y | Y | N | Y | Y | Y | C | Y | 6 |
| 101 | Greenhalgh 2015 | N | Y | Y | Y | N | Y | Y | Y | C | Y | 7 |
| 102 | Poonawalla 2015 | N | Y | C | Y | N | Y | Y | Y | C | N | 5 |
| 103 | Geng 2015 | Y | C | Y | Y | N | Y | Y | Y | C | N | 6 |
| 104 | Roze 2015 | Y | C | Y | Y | N | Y | Y | Y | C | Y | 7 |
| 105 | Wang 2015 | N | C | Y | Y | N | Y | Y | N | C | N | 4 |
| 106 | Peng 2015 | N | N | Y | Y | N | Y | Y | Y | C | N | 5 |
| 107 | Mistro 2016 | N | C | Y | Y | N | Y | Y | Y | C | Y | 6 |
| 108 | Liberato 2016 | N | N | Y | Y | N | Y | Y | Y | C | N | 5 |
| 109 | Nerich 2016 | N | C | N | N | N | Y | Y | Y | C | Y | 4 |
| 110 | Loveman 2016 | Y | Y | Y | Y | Y | Y | Y | N | C | Y | 8 |
| 111 | Chhatwal 2016 | N | Y | Y | Y | N | Y | Y | Y | C | N | 6 |
| 112 | Chit 2016 | N | Y | Y | Y | N | Y | Y | Y | C | Y | 7 |
| 113 | Ben Hadj Yahia 2016 | N | N | Y | Y | N | Y | Y | N | C | N | 4 |
| 114 | Jones-Hughes 2016 | Y | C | Y | Y | N | Y | Y | N | C | Y | 6 |
| 115 | Archer 2016 | Y | N | Y | Y | N | Y | Y | N | C | Y | 6 |
| 116 | Kourlaba 2016 | N | Y | Y | Y | N | Y | Y | N | C | N | 5 |
| 117 | Vellopoulou 2016 | N | Y | Y | Y | N | Y | Y | N | C | Y | 6 |
| 118 | Bridle 2016 | Y | Y | Y | Y | N | Y | Y | N | C | N | 6 |
| 119 | Ahmadiani 2016 | N | N | Y | Y | N | Y | Y | N | C | N | 4 |
| 120 | Dretzke 2011 | Y | Y | Y | Y | N | Y | Y | N | C | N | 6 |
| 121 | Men 2016 | N | C | Y | Y | N | Y | Y | N | C | N | 4 |
| 122 | Johnston 2017 | Y | Y | N | N | N | Y | Y | Y | C | Y | 6 |
| 123 | Bunchai 2017 | N | Y | Y | Y | N | Y | Y | Y | C | Y | 7 |
| 124 | Ding 2017 | N | N | N | N | N | Y | Y | C | C | N | 2 |
| 125 | Pike 2017 | N | Y | Y | Y | N | Y | Y | Y | C | Y | 7 |
| 126 | Torres 2017 | Y | Y | Y | Y | Y | Y | Y | Y | Y | Y | 10 |
| 127 | Corbett 2017 | Y | Y | Y | Y | Y | Y | Y | Y | Y | Y | 10 |
| 128 | Kotrium 2017 | N | N | N | N | Y | Y | Y | C | C | N | 3 |
| 129 | Boer 2017 | N | N | N | N | N | Y | Y | C | C | Y | 3 |
| 130 | Didik 2017 | N | Y | N | N | N | Y | Y | Y | Y | Y | 6 |
| 131 | Wijnen 2017 | Y | Y | Y | Y | N | Y | Y | Y | Y | Y | 9 |
| 132 | Ahmad 2018 | N | Y | Y | Y | N | Y | Y | Y | C | N | 6 |
| 133 | Anees 2018 | N | Y | N | N | N | Y | Y | C | C | Y | 4 |
| 134 | Sophy 2018 | N | Y | Y | N | N | Y | Y | Y | C | N | 5 |
| 135 | Li 2018 | N | N | N | N | N | Y | Y | C | C | N | 2 |
| 136 | Zhao 2018 | N | N | N | N | N | Y | Y | C | C | N | 2 |
| 137 | Andronis 2017 | N | Y | Y | C | Y | Y | Y | Y | Y | Y | 8 |
| 138 | Le 2017 | N | Y | Y | C | Y | Y | Y | Y | Y | Y | 8 |
| 139 | Herzog 2017 | Y | Y | Y | C | Y | Y | Y | Y | Y | Y | 9 |
| 140 | Iannazzo 2017 | N | Y | Y | Y | Y | Y | Y | Y | Y | N | 8 |
| 141 | Camacho 2018 | Y | Y | Y | C | Y | Y | Y | Y | Y | Y | 9 |
| 142 | Castro 2018 | Y | Y | Y | C | Y | Y | Y | Y | Y | Y | 9 |
| 143 | Angiolella 2018 | N | Y | Y | C | Y | Y | Y | Y | Y | Y | 8 |
| 144 | Elshout 2018 | N | Y | Y | C | Y | Y | Y | Y | Y | N | 7 |
| 145 | Gedge 2018 | N | Y | N | C | Y | Y | N | N | Y | Y | 5 |
| 146 | Grochtdreis 2018 | N | Y | Y | C | Y | Y | Y | Y | Y | N | 7 |
| 147 | Han 2018 | N | Y | Y | C | Y | Y | Y | Y | Y | Y | 8 |
| 148 | Hsiao 2018 | N | Y | Y | C | Y | Y | Y | Y | Y | Y | 8 |
| 149 | Hui 2018 | Y | Y | Y | C | Y | Y | Y | Y | Y | Y | 9 |
| 150 | Jafari 2018 | N | Y | Y | C | Y | Y | Y | Y | Y | N | 7 |
| 151 | Jean 2018 | N | Y | N | C | Y | Y | N | N | Y | Y | 5 |
| 152 | Kromer 2018 | N | Y | Y | Y | Y | Y | Y | Y | Y | Y | 9 |
| 153 | Le 2018 | N | Y | Y | C | Y | Y | Y | Y | Y | N | 7 |
| 154 | Leonart 2018 | N | Y | Y | Y | Y | Y | Y | Y | Y | Y | 9 |
| 155 | Mcqueen 2018 | N | Y | N | C | Y | Y | Y | Y | Y | N | 6 |
| 156 | Ng 2018 | N | Y | Y | Y | Y | Y | Y | Y | Y | Y | 9 |
| 157 | Nishikawa 2018 | N | Y | Y | C | Y | Y | N | N | Y | Y | 6 |
| 158 | Petrou 2018 | N | Y | Y | C | Y | Y | N | N | Y | Y | 6 |
| 159 | Puig-juNy 2018 | N | Y | Y | C | Y | Y | N | N | Y | Y | 6 |
| 160 | Rodriguez-martinez 2018 | N | Y | Y | C | Y | Y | Y | Y | Y | Y | 8 |
| 161 | Silas 2018 | N | Y | Y | C | Y | Y | N | N | Y | Y | 6 |
| 162 | Teoh 2018 | N | Y | Y | C | Y | Y | Y | Y | Y | Y | 8 |
| 163 | Verma 2018 | N | Y | N | C | Y | Y | Y | Y | Y | Y | 8 |
| 164 | Wortman2018 | Y | Y | Y | C | Y | Y | N | N | Y | N | 6 |
| 165 | You 2018 | N | Y | Y | C | Y | Y | Y | Y | Y | Y | 8 |
| Item 1: Was an 'a priori' design provided?  Item 2: Was there duplicate study selection and data extraction?  Item 3: Was a comprehensive literature search performed?  Item 4: Was the status of publication (i.e. grey literature) used as an inclusion criterion?  Item 5: Was a list of studies (included and excluded) provided?  Item 6: Were the characteristics of the included studies provided?  Item 7: Was the scientific quality of the included studies assessed and documented?  Item 8: Was the scientific quality of the included studies used appropriately in formulating conclusions?  Item 9: Were the methods used to combine the findings of studies appropriate?  Item 10: Was the conflict of interest included?  Y-yes; N-no; C-Can not answer | | | | | | | | | | | | |
