# Supplementary material for: An overview of the characteristics and quality assessment criteria in systematic review of pharmacoeconomics
Source: PLoS One. 2021 Feb 8;16(2):e0246080. doi: 10.1371/journal.pone.0246080 (PMC7870091; doi:10.1371/journal.pone.0246080)
Supplement: Attachment — Submitted filename: Response to Reviewers.docx [file pone.0246080.s006.docx]

Dear editor and reviewers,

Thank you for giving us the opportunity to revise the manuscript again. We have made required changes to the paper according to the comments and gave a point-by-point response below. We have checked the grammatical errors or typos, which caused minor changes throughout the main document. We have highlighted all the changes in the revised manuscript with tracked changes. We hope that the revised version and our responses have a better chance of being accepted by your journal.

Review Comments to the Author

Reviewer #1: Thanks to the authors for addressing the previous review comments well. However, there are some minor revisions that need to be made in the manuscript.

Comment 1: There are several grammatical errors or typos in the article, and I have listed some of them below. It would be great if the authors could proofread the article carefully and correct the errors.

1) Abstract background section: “A systematic review of economic evaluations plays a critical…..” to “The systematic reviews of economic evaluations plays a critical…..”

2) Abstract results section: “Others include the Quality of Health Economic Studies (QHES)” to “Others included the Quality of Health Economic Studies (QHES)”

3) Discussion: “…… these pharmcoeconomic evaluation tools.” to “…... these pharmacoeconomic evaluation tools.”

Response: Many thanks for these useful and valid suggestions!

We have corrected the errors in the article. Please see the revision of the manuscript.

Comment 2: Please spell out the name of the database described in the methods section of the abstract.

Response: Many thanks for these useful and valid suggestions!

We have detailed the name of the database described in the methods section of the abstract as following:

“Nine English and Chinese databases including the Cochrane Library, PubMed, EMbase (Ovid), NHS economic evaluation database (NHSEED) (Ovid), Health Technology Assessment (HTA) database, Chinese National Knowledge Infrastructure (CNKI), WangFang, VIP Chinese Science & Technology Periodicals (VIP) and Chinese Biomedical Literature Database (CBM) were searched.”
